# Radiation safety and cancer risks in pediatric computed tomography: a systematic review of large-scale cohort studies

**DOI:** 10.3389/fped.2026.1885951

**Published:** 2026-07-31

**Authors:** V. M. Almatova, K. S. Absattarova, I. M. Bozheyeva, G. L. Medetova, A. M. Gabbassova, K. K. Toguzbaeva

**Affiliations:** 1 Scientific Research Institute of Radiology named after Zh. H. Khamzabayev, Astana Medical University, Astana, Kazakhstan; 2 Department of Public Health, Kazakh National Medical University Named After S.D. Asfendiyarov, Almaty, Kazakhstan

**Keywords:** brain tumors, cancer risk, dose-response, leukemia, pediatric computed tomography, radiation exposure, radiation safety

## Abstract

**Background:**

The increasing use of computed tomography (CT) in pediatric populations has raised concerns regarding radiation-induced malignancies due to higher radiosensitivity and longer post-exposure lifespan in children.

**Objective:**

This study aimed to synthesize evidence from large-scale cohort studies on the association between pediatric CT exposure and subsequent cancer risk, with a focus on leukemia and brain tumors.

**Methods:**

A systematic review was conducted following PRISMA 2020 guidelines. Literature searches were performed in PubMed and the Cochrane Library from database inception to 30 June 2025. Seven large cohort studies, including more than 11 million individuals from Europe, Asia, and Australia, were included. Data on radiation exposure, cancer outcomes, follow-up, effect measures, and methodological quality were extracted independently by two reviewers.

**Results:**

A consistent dose-response relationship was observed across studies. Excess relative risk (ERR) per mGy ranged from 0.033 to 0.045 for leukemia and from 0.006 to 0.023 for brain tumors in studies reporting organ-dose estimates. The risk was higher in children exposed at younger ages, particularly under 5 years, and increased with cumulative exposure. Scan-count analyses also suggested higher incidence with repeated CT, although scan count is a proxy for dose and should not be interpreted as equivalent to organ-dose ERR. Although indication bias was identified, adjusted and lagged analyses generally supported a persistent association.

**Conclusion:**

Pediatric CT exposure is associated with a small but measurable increase in long-term cancer risk. These findings should be interpreted alongside the diagnostic benefit of CT in trauma, oncology, and life-threatening illness. Clinically justified CT remains essential; the results support strict justification, dose optimization, and the use of non-ionizing alternatives when they can answer the clinical question.

## 1 Introduction

The use of computed tomography (CT) in pediatric populations has increased substantially over recent decades, primarily due to its high diagnostic accuracy and rapid acquisition time (1, 2). Despite these advantages, concerns persist regarding the potential long-term effects of ionizing radiation, particularly in children, who are more radiosensitive and have a longer life expectancy during which radiation-induced malignancies may develop (3, 4).

Large epidemiological studies have reported associations between CT-related radiation exposure and increased risks of leukemia and brain tumors in children (5, 6). These findings are supported by landmark cohort studies conducted in the United Kingdom and Australia, which demonstrated a clear dose–response relationship even at relatively low levels of radiation exposure (5, 6).

The observed associations are consistent with risk estimates derived from studies of atomic bomb survivors, which remain the most comprehensive source of evidence on radiation-induced cancer and provide a biological basis for understanding low-dose radiation effects (7, 8).

However, interpretation of these findings is complicated by several methodological challenges, including confounding by indication, variability in radiation dose estimation, and differences in follow-up duration across studies (9, 10). In addition, more recent multinational cohort studies have expanded the evidence base by incorporating diverse populations and improved dosimetric modeling techniques (11, 12).

At the same time, the magnitude of cancer risk associated with low-dose radiation exposure remains a subject of ongoing scientific debate, particularly in relation to the linear no-threshold (LNT) model (13, 14). Given the increasing global use of CT imaging, reassessment of radiation-related risks in the context of modern clinical practice and radiation protection strategies is essential (15).

Although numerous individual cohort studies and narrative reviews have addressed this topic, there remains a lack of focused synthesis specifically integrating large-scale epidemiological evidence using consistent risk metrics. In particular, comparative evaluation of excess relative risk (ERR), age-related susceptibility, and cumulative exposure effects has not been sufficiently systematized.

Therefore, the aim of this study was to systematically review and synthesize evidence from large-scale cohort studies on radiation exposure from pediatric CT and subsequent cancer risk, with a particular focus on leukemia and brain tumors, and to provide clinically relevant insights for optimizing radiation safety in pediatric imaging.

## 2 Materials and methods

### 2.1 Study design

This study was conducted as a systematic review in accordance with the Preferred Reporting Items for Systematic Reviews and Meta-Analyses (PRISMA 2020) guidelines (16) and the Cochrane Handbook for Systematic Reviews of Interventions.

The review question was structured using the PICO framework: population, children and adolescents exposed to CT before adulthood; exposure, ionizing radiation from diagnostic CT; comparator, unexposed children, lower-dose groups, or lower scan-count categories; outcomes, leukemia, brain/CNS tumors, all cancers, and other reported malignancies; study design, cohort or large-scale registry-based observational studies. The protocol was not prospectively registered in PROSPERO, which is now stated as a limitation.

### 2.2 Search strategy

A comprehensive literature search was performed in PubMed and the Cochrane Library from database inception to 30 June 2025. The search strategy combined Medical Subject Headings (MeSH) and free-text terms related to pediatric computed tomography and radiation-associated cancer risk.

The search was restricted to PubMed/MEDLINE and the Cochrane Library because these databases were selected *a priori* as the principal biomedical sources for the review. Embase, Scopus, and Web of Science were not researched; this limitation is acknowledged because relevant conference records or recently indexed studies may have been missing.

The complete PubMed search string was: [“Tomography, x-Ray Computed”(MeSH) OR “computed tomography” OR “CT scan*” OR “diagnostic imaging”] AND [“Child”[MeSH] OR “Infant”[MeSH] OR “Adolescent”[MeSH] OR child* OR pediatric* OR paediatric* OR adolescent*] AND [“Radiation Dosage”(MeSH) OR radiation OR ionizing OR dose OR mGy OR “organ dose”] AND [“Neoplasms”(MeSH) OR cancer OR malignan* OR leukemia OR leukaemia OR lymphoma OR “brain tumor*” OR “brain tumour*” OR CNS]. The Cochrane Library search used the same concept blocks with simplified keyword syntax.

Additionally, reference lists of relevant articles were manually screened to identify further eligible studies.

Pooled analyses and reports with overlapping cohorts were used for contextual interpretation but were not counted as additional primary studies in the main tables to avoid double counting participants and risk estimates from the same underlying cohorts.

### 2.3 Eligibility criteria

Studies were included if they met the following criteria:

1. involved pediatric populations (individuals <18 years of age);
2. assessed exposure to CT-related ionizing radiation;
3. reported cancer outcomes (e.g., leukemia, brain tumors, or other malignancies);
4. used cohort or large-scale observational designs;
5. provided quantitative risk estimates (e.g., ERR, IRR, RR, HR, or SIR).

Studies were excluded if they:

1. included only adult populations;
2. did not assess CT-related radiation exposure;
3. lacked cancer outcome data;
4. were case reports, editorials, or narrative reviews.

When multiple publications used overlapping populations, the primary cohort report or most directly relevant outcome-specific report was retained for the main synthesis; overlapping pooled analyses were discussed narratively.

### 2.4 Study selection

All records identified through database searching were independently screened based on titles and abstracts. Full-text articles were assessed for eligibility according to the predefined inclusion criteria. Discrepancies were resolved through discussion among the authors.

**Figure 1:**
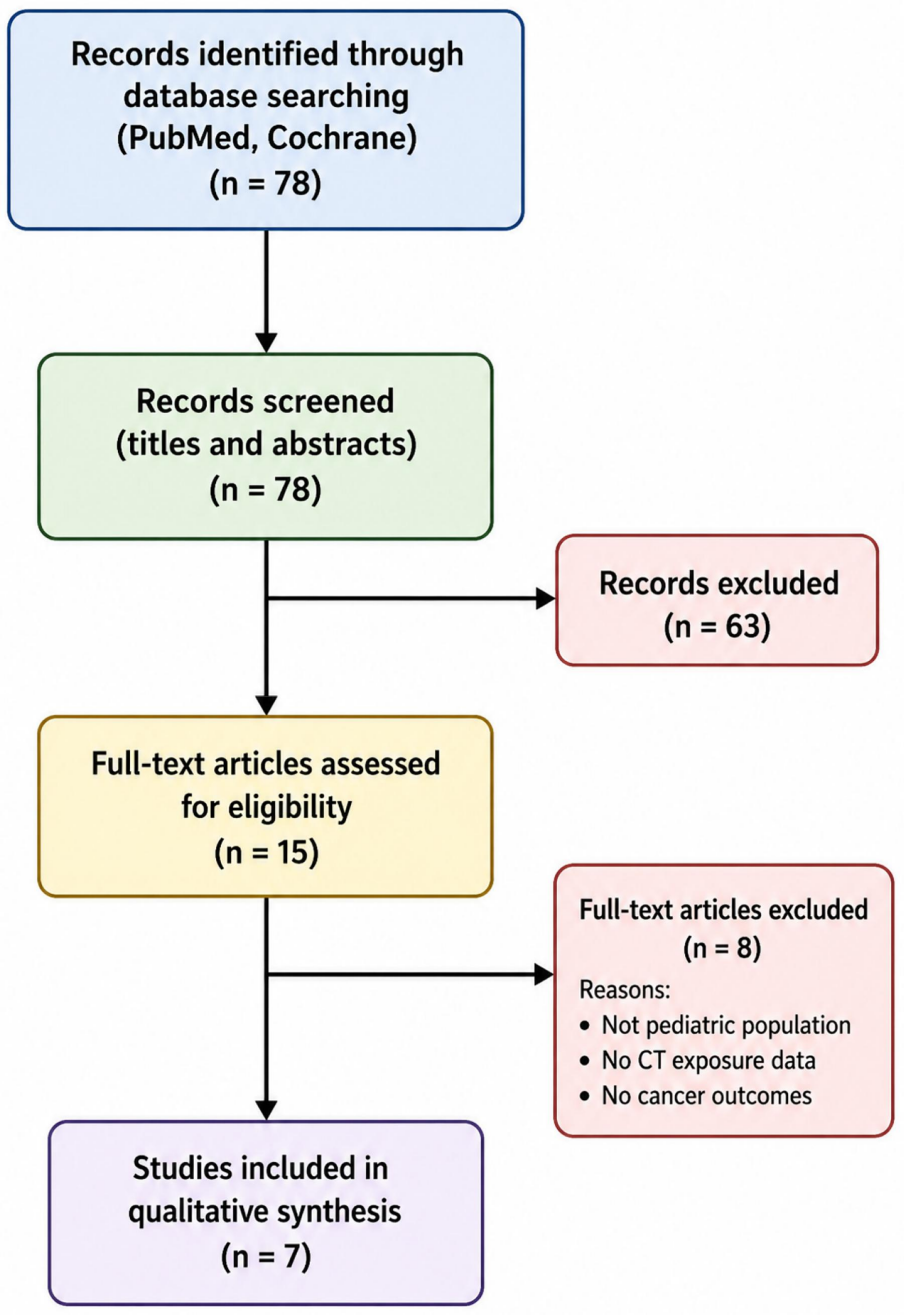
PRISMA flow diagram of study selection process.

A total of 78 records were identified, of which 15 full-text articles were assessed for eligibility. Seven primary cohort studies met the inclusion criteria and were included in the final analysis. Full-text exclusions were due to non-pediatric populations, no CT-specific exposure data, absence of cancer outcome data, review design, or overlapping cohort reports. The study selection process is presented in the PRISMA flow diagram (Figure 1).

### 2.5 Data extraction

Data was extracted independently by two reviewers using a standardized form. Discrepancies were resolved by consensus and, when necessary, by consultation with a third author. The following variables were collected:

- study characteristics (country, study period, sample size);
- patient demographics (age at exposure);
- type and number of CT examinations;
- radiation dose estimates;
- follow-up duration;
- cancer outcomes;
- risk estimates (ERR, IRR, RR, HR, SIR).

### 2.6 Risk of bias assessment

The methodological quality of included cohort studies was assessed using the Newcastle–Ottawa Scale (NOS) for observational studies (17). Each study was evaluated across three domains: selection of participants, comparability of study groups, and outcome assessment.

Studies scoring 7–9 points were considered high quality, 4–6 moderate quality, and ≤3 low quality. Risk of bias assessment was performed independently by two reviewers, with disagreements resolved by consensus. Supplementary Table S1 presents the Newcastle-Ottawa Scale (NOS) assessment for each included study.

NOS ratings were not used to exclude studies; they informed the interpretation of certainty, especially for comparability and outcome ascertainment domains.

### 2.7 Data synthesis

Due to heterogeneity in study design, exposure assessment methods, latency restrictions, cancer outcome definitions, and reported effect measures, a quantitative meta-analysis was not performed. Instead, a qualitative synthesis was conducted, focusing on dose-response relationships, age-dependent risk, cumulative exposure effects, and whether estimates changed after adjustment for indication bias or exclusion of early follow-up.

Findings were summarized across studies with emphasis on consistency of risk estimates and methodological differences.

A quantitative meta-analysis was judged inappropriate because the studies differed in the organ dose modeled, lag period, comparator definition, cancer registry linkage, and effect measure. Pooling RR, HR, IRR, SIR, and ERR estimates would risk producing a summary estimate that is statistically precise but epidemiologically misleading.

### 2.8 Ethical considerations

Ethical approval was not required, as this study is based on previously published data and does not involve human participants or identifiable personal data.

## 3 Results

### 3.1 Study characteristics

A total of seven large-scale epidemiological cohort studies were included in this review, encompassing over 11 million pediatric patients from multiple countries, including the United Kingdom, Australia, Japan, Taiwan, Germany, France, and the Netherlands.

Sample sizes varied substantially, ranging from 763 participants to over 10 million individuals. The age at exposure generally included children and adolescents up to 18–22 years. Follow-up was reported differently across studies as mean, median, maximum, or lagged study-specific follow-up, ranging from approximately 2–10 years. The proportion of children undergoing a single CT examination ranged widely across cohorts.

A detailed summary of study characteristics is presented in Table 1.

**Table 1:** Characteristics of included cohort studies.

No	Study	Country	Study period	Sample size	Age range (years)	Follow-up (years)	Single CT (%)
1	Shibata et al. (18)	Japan	2013	763	0–19	5	33.9
2	Pearce et al. (5)	UK	1985–2002	178,604	0–22	10	71.0
3	Mathews et al. (6)	Australia	1985–2005	10,939,680	0–19	9.5	82.0
4	Huang et al. (19)	Taiwan	1996–2008	122,086	0–18	5	93.4
5	Meulepas et al. (11)	Netherlands	1979–2012	168,394	0–18	10	64.2
6	Journy et al. (9)	France	2000–2010	67,274	0–10	4	92.0
7	Krille et al. (20)	Germany	1980–2010	39,184	0–15	2	70.0

CT, computed tomography. Follow-up is reported as mean, median, maximum, or study-specific lagged follow-up according to the original publication; therefore, values should be compared qualitatively rather than as identical follow-up metrics.

### 3.2 CT utilization and exposure patterns

Across the included studies, head CT scans represented the majority of examinations, accounting for approximately 57% to 100% of all scans. In several cohorts, more than 60% of imaging procedures involved the brain.

The distribution of CT scans across anatomical regions varied, with lower proportions observed for thoracic, abdominal, and musculoskeletal imaging. These differences reflect variability in clinical indications and healthcare practices across countries (Table 2).

**Table 2:** Distribution of CT scans by anatomical regions.

Study	Total CTs	Brain/Head/Neck (%)	Thorax (%)	Abdomen (%)	Bone/Soft tissue (%)
Shibata et al. (18)	1,033	31.6	17.6	16.9	13.6
Pearce et al. (5)	283,919	64.0	7.0	9.0	–
Mathews et al. (6)	608,211	59.4	1.7	5.0	9.5
Huang et al. (19)	28,185	100.0	–	–	–
Meulepas et al. (11)	262,227	65.5	8.1	7.6	8.3
Journy et al. (9)	219,544	56.9	23.1	9.1	1.7
Krille et al. (20)	63,214	72.0	9.2	3.3	5.1

CT, computed tomography; mGy, milligray; ERR, excess relative risk; IRR, incidence rate ratio; SIR, standardized incidence ratio.

### 3.3 Dose–response relationship

Most included studies demonstrated a positive dose-response relationship between CT-related radiation exposure and cancer risk, but the strength of evidence varied by outcome, latency restriction, and dose-reconstruction method.

For leukemia, excess relative risk (ERR) per mGy ranged from 0.033 to 0.045 in studies that reported organ-dose ERR estimates. Findings from major cohorts in the United Kingdom and Australia were broadly consistent, although direct numerical comparison is limited by differences in dose reconstruction and latency assumptions.

For brain tumors, ERR values ranged from 0.006 to 0.023 per mGy where reported. Although lower in magnitude compared with leukemia, the association remained evident across multiple studies, particularly with longer follow-up periods; however, confounding by indication is especially relevant for this outcome.

A comparative summary of risk estimates across studies is presented in Table 3.

**Table 3:** Risk estimates for brain tumors and overall cancer across studies.

**Study**	**Metric**	**Brain/CNS tumors (estimate, 95% CI)**	**All cancers (estimate, 95% CI)**
Pearce et al. (5)	RR	2.82 (1.33–6.03)	–
Mathews et al. (6)	IRR	2.13 (1.88–2.41)	1.24 (1.20–1.29)
Huang et al. (19)	HR	2.56 (1.44–4.54)	2.43 (0.88–6.68)
Meulepas et al. (11)	SIR	2.05 (1.48–2.83)	1.48 (1.34–1.61)
Journy et al. (9)	ERR	0.012 (−0.013–0.037)	–
Krille et al. (20)	SIR	1.35 (0.54–2.78)	1.82 (1.29–2.50)

CT, computed tomography; mGy, milligray; ERR, excess relative risk; IRR, incidence rate ratio; SIR, standardized incidence ratio.

Because Table 3 includes different epidemiological measures (RR, HR, IRR, SIR, and ERR), the values are presented to show direction and consistency of association within each original study. They should not be read as directly interchangeable effect sizes. Outcome definitions also varied across studies; therefore, each estimate should be interpreted with reference to the outcome and measure reported in the original publication.

### 3.4 Cancer risk and incidence

Leukemia was the most consistently reported outcome and showed the strongest association with radiation exposure. Several studies demonstrated a statistically significant increase in leukemia incidence with increasing radiation dose, particularly in patients receiving cumulative doses exceeding 30 mGy.

Brain tumors were the second most frequently reported outcome. While the magnitude of risk was lower than for leukemia, dose–response patterns remained evident. Risk estimates varied across studies due to differences in methodology and follow-up duration.

Overall cancer incidence was also elevated in exposed populations. In large cohort studies, the incidence rate ratio (IRR) for all cancers was approximately 1.24, indicating a 24% increase compared to non-exposed groups.

Despite these relative increases, the absolute excess risk remained low. Published estimates suggest approximately one additional cancer case per 500–2,000 pediatric CT scans in selected scenarios, depending on age, sex, anatomical region, organ dose, and latency assumptions (5, 21). These values should be interpreted as study- and scenario-specific estimates rather than generalized risks for every pediatric CT examination.

The total number of reported cancer cases across studies is summarized in Table 4.

**Table 4:** Summary of cancer cases reported across included studies.

Study	All cancers	Leukemia	Brain tumors
Shibata et al. (18)	67	10	8
Pearce et al. (5)	–	74	135
Mathews et al. (6)	3,159	211	283
Huang et al. (19)	122	17	30
Meulepas et al. (11)	454	44	84
Journy et al. (9)	106	25	27
Krille et al. (20)	38	12	7

### 3.5 Modifying factors

Several factors influenced the relationship between CT exposure and cancer risk.

Age at exposure was a key determinant, with the highest risks observed in children under 5 years of age. Risk decreased with increasing age at exposure.

Cumulative exposure was also associated with increased risk. In scan-count analyses, each additional CT examination was associated with an approximate increase of 0.16 in IRR in the original registry-based report; however, this estimate should be interpreted as study-specific and as a proxy for cumulative dose rather than a generalizable dose-response coefficient.

Sex differences were reported in some studies, with slightly higher risks observed in females for certain cancer types, particularly solid tumors.

### 3.6 Latency and temporal patterns

Cancer risk varied depending on the latency period following exposure. Increased incidence was observed as early as 1–4 years after CT exposure.

Across latency restrictions of 1, 5, and 10 years, overall cancer incidence was generally higher among exposed populations in studies that reported lagged analyses, but the magnitude attenuated after longer lag periods in some studies. These latency-specific findings should therefore be interpreted in relation to the original study design and outcome definition rather than as generalized pooled estimates.

Risk estimates for leukemia were highest within the first 5 years, while brain tumor risk remained elevated over longer follow-up periods.

Radiation dose characteristics and estimation methods are summarized in Table 5.

**Table 5:** Radiation dose characteristics across included studies.

**Study**	**CT type**	**Estimated dose per scan (mGy)**	**Cumulative dose (mGy)**	**Dose estimation method**
Shibata et al. (18)	Head CT	20–30	Not reported	Dosimetry reconstruction
Pearce et al. (5)	Head CT	∼30	50–60	Organ dose modeling
Mathews et al. (6)	Multiple	5–60	Up to 100+	Registry-based estimation
Huang et al. (19)	Head CT	∼20	∼40	Dosimetry reconstruction
Meulepas et al. (11)	Multiple	10–50	Not reported	Modeling and cohort data
Journy et al. (9)	Head CT	∼25	Not reported	Organ dose modeling
Krille et al. (20)	Multiple	5–40	Variable	Retrospective assessment

### 3.7 Indication bias and confounding

A major methodological issue identified across studies was confounding by indication. In some cases, CT scans were performed due to early symptoms of undiagnosed malignancies, particularly slow-growing brain tumors.

Adjustment for this bias reduced risk estimates in several studies; however, associations between CT exposure and cancer risk generally remained statistically significant.

Reverse causation was particularly relevant for brain tumors because early neurological symptoms may prompt CT before diagnosis. Studies that applied lag periods or excluded patients with cancer shortly after imaging generally reported attenuated but persistent associations, suggesting that both indication bias and radiation-related risk may contribute.

Across major cohorts, approaches to indication bias differed: some studies applied lag periods, excluded cancers diagnosed shortly after CT, adjusted for predisposing conditions where registry information was available, or performed sensitivity analyses for patients with conditions associated with cancer susceptibility. These methods reduce but cannot fully eliminate confounding by indication, particularly for brain tumors and other outcomes for which early symptoms may trigger diagnostic imaging.

### 3.8 Summary of findings

Overall, the included studies demonstrate consistent evidence of an association between pediatric CT exposure and increased cancer risk, particularly for leukemia and brain tumors.

The relationship is characterized by a dose–response pattern, higher susceptibility at younger ages, and increasing risk with cumulative exposure. Although absolute risks remain low, the widespread use of CT imaging in children suggests a meaningful public health impact.

## 4 Discussion

This systematic review synthesizes evidence from large-scale cohort studies examining the association between pediatric CT exposure and subsequent cancer risk. Across diverse populations and healthcare systems, the findings consistently demonstrate a dose–response relationship between ionizing radiation from CT and the risk of malignancies, particularly leukemia and brain tumors.

The observed excess relative risk estimates for leukemia (0.033–0.045 per mGy) and brain tumors (0.006–0.023 per mGy) are broadly consistent with previously reported data from both medical and non-medical radiation exposure settings, including the Life Span Study of atomic bomb survivors (7, 8). However, the reviewed studies used different dose metrics and effect measures, so ERR, RR, IRR, HR, and SIR estimates should not be interpreted as directly comparable. This consistency supports the biological plausibility of radiation-induced carcinogenesis even at relatively low doses while preserving appropriate caution about numerical synthesis.

One of the key findings of this review is the strong influence of age at exposure. Children exposed at younger ages, particularly under 5 years, demonstrated significantly higher risks compared to older age groups. This is in line with established radiobiological principles, which indicate increased radiosensitivity of developing tissues (4). In addition, cumulative exposure emerged as a critical determinant of risk, with repeated CT examinations contributing to a measurable increase in cancer incidence.

Despite these consistent associations, interpretation of the results requires careful consideration of methodological limitations. A major concern across studies is confounding by indication, particularly for brain tumors. CT scans are often performed due to early symptoms of undiagnosed malignancies, which may lead to overestimation of risk. However, several studies have demonstrated that adjustment for this bias reduces but does not eliminate the association, suggesting that the observed relationships cannot be fully explained by reverse causation alone (9, 10).

Another important consideration is variability in radiation dose estimation. Differences in CT protocols, scanner technology, and dosimetric modeling approaches introduce heterogeneity across studies. Although this limits direct comparability, the consistency of dose–response patterns across independent cohorts strengthens the overall evidence base (11, 12).

Recent pooled and multinational analyses provide useful contextual evidence, but many draw from overlapping underlying cohorts. They were therefore discussed narratively rather than added as separate primary studies in the main tables, to avoid double counting the same exposed populations.

The 2023 joint analysis of several pediatric CT cohorts (12) supports the direction of association observed in earlier UK and Australian cohorts and provides more precise pooled evidence for leukemia and brain tumor risks. Because such analyses partially reuse participants from earlier national cohorts, they were used to contextualize the findings rather than added as independent rows in the main evidence tables.

From a risk perspective, previously published estimates suggest that the lifetime attributable risk of cancer following pediatric CT exposure may range from approximately 1 case per 500–2,000 scans, depending on age and anatomical region (5, 21). Younger children appear to have higher estimated risks compared to adolescents, reflecting increased radiosensitivity.

The linear no-threshold (LNT) model remains a pragmatic basis for radiation protection because it assumes that cancer risk increases linearly with dose without a safe threshold at low doses. ICRP, UNSCEAR, and BEIR VII (22–24) support using LNT-based approaches for population-level protection and optimization, while acknowledging statistical uncertainty at very low individual doses and the need to avoid interpreting LNT as a precise prediction for a single child. In clinical practice, this supports justification and dose optimization rather than avoidance of necessary CT examinations.

Although these risks are small relative to the baseline lifetime cancer risk in the general population, they remain clinically relevant given the widespread use of CT in pediatric practice.

In addition to oncological outcomes, emerging evidence has suggested potential associations between cumulative radiation exposure and non-cancer outcomes, including cardiovascular disease; however, these findings remain limited and require further investigation.

From a clinical perspective, the absolute risk associated with pediatric CT remains low. However, given the widespread use of CT in pediatric populations, even small increases in individual risk may translate into a significant public health impact (19).

At the same time, CT provides substantial diagnostic benefits when rapid and accurate imaging is needed, including trauma, suspected intracranial pathology, cancer staging or surveillance, and acute life-threatening conditions. The objective of radiation protection is therefore not to avoid clinically indicated CT, but to avoid low-value or repeated examinations and to tailor protocols to the child's size and clinical question.

These findings underscore the importance of strict adherence to radiation protection principles, including justification and optimization of imaging procedures. The ALARA principle remains central to minimizing unnecessary exposure, and alternative imaging modalities such as ultrasound and magnetic resonance imaging should be considered whenever clinically appropriate (15).

Modern optimization strategies include pediatric-size-adjusted protocols, automated exposure control, iterative or model-based reconstruction, diagnostic reference levels, protocol audit, and decision-support tools to reduce repeated or low-value CT imaging.

The present review has several strengths. It focuses specifically on large-scale cohort studies with robust epidemiological design and includes data from multiple countries, enhancing generalizability. In addition, the synthesis of consistent risk metrics, such as ERR and IRR, allows for meaningful comparison across studies.

However, limitations should also be acknowledged. The number of eligible primary cohort studies was limited, and heterogeneity in study design, indication data, organ-dose reconstruction, latency restrictions, outcome definitions, and effect measures precluded a formal meta-analysis. Restriction to PubMed and the Cochrane Library may have missed studies indexed elsewhere, including Embase, Scopus, and Web of Science, and publication bias could not be formally assessed because the number of comparable estimates was small. Furthermore, potential residual confounding, reverse causation, incomplete clinical indication data, and variability in follow-up duration may influence risk estimates. Future research should focus on harmonized exposure definitions, standardized outcome definitions, transparent reporting of effect measures, and longer follow-up.

The review protocol was not prospectively registered in PROSPERO. This reduces transparency compared with a registered systematic review and should be considered when interpreting the certainty of the synthesis.

## 5 Conclusion

This systematic review of large-scale cohort studies demonstrates that exposure to ionizing radiation from computed tomography in childhood is associated with a small but measurable increase in the risk of malignancies, particularly leukemia and brain tumors. A consistent dose–response relationship was observed, with higher risks among younger children and those exposed to multiple scans.

Although the absolute risk remains low, the widespread use of CT in pediatric populations underscores the importance of minimizing unnecessary radiation exposure. These findings reinforce the need for strict clinical justification of CT examinations, optimization of imaging protocols in accordance with the ALARA principle, and increased use of non-ionizing alternatives such as ultrasound and magnetic resonance imaging when appropriate.

Future research should focus on harmonized organ-dose reconstruction, longer follow-up, transparent indication data, absolute-risk reporting, and refined low-dose radiation risk models in children.

From a clinical perspective, CT examinations in pediatric patients should be carefully justified, with particular attention to minimizing repeated exposures. Younger children require stricter dose optimization strategies due to increased radiosensitivity. The anatomical region of imaging should also be considered, as it is often associated with site-specific cancer risk. Whenever feasible, alternative imaging modalities that do not involve ionizing radiation should be prioritized.

## Data Availability

The original contributions presented in the study are included in the article/Supplementary Material, further inquiries can be directed to the corresponding author.
