# Supplementary material for: Radiation safety and cancer risks in pediatric computed tomography: a systematic review of large-scale cohort studies
Source: Front Pediatr. 2026 Jul 31;14:1885951. doi: 10.3389/fped.2026.1885951 (PMC13473082; doi:10.3389/fped.2026.1885951)
Supplement: Supplementary file 1 [file Table1.docx]

**PRISMA 2020 Checklist**

Checklist prepared for the revised systematic review manuscript. Locations refer to manuscript sections, tables, figures, and supplementary material in the revised submission.

| **PRISMA item** | **Topic** | **Location** | **Where addressed / note** |
| --- | --- | --- | --- |
| 1 | Title | Title | Title identifies the article as a systematic review of large-scale cohort studies. |
| 2 | Abstract | Abstract | Structured abstract reports background, objective, methods, results, and conclusion. |
| 3 | Rationale | Introduction | Rationale for reviewing pediatric CT-related radiation exposure and cancer risk is described. |
| 4 | Objectives | Introduction; Section 2.1 | Review objective and PICO framework are stated. |
| 5 | Eligibility criteria | Section 2.3 | Inclusion and exclusion criteria define population, exposure, outcomes, study design, and publication type. |
| 6 | Information sources | Section 2.2 | PubMed/MEDLINE and Cochrane Library were searched from database inception to 30 June 2025; database restriction is acknowledged in Limitations. |
| 7 | Search strategy | Supplementary Material: Complete Search Strategy | Full PubMed/MEDLINE search string and equivalent Cochrane Library keyword strategy are provided. |
| 8 | Selection process | Section 2.4; Figure 1 | Title/abstract and full-text screening are described; PRISMA flow diagram reports 78 records, 15 full-text articles assessed, and 7 included studies. |
| 9 | Data collection process | Section 2.5 | Independent data extraction by two reviewers and consensus resolution are described. |
| 10a | Data items: outcomes | Section 2.5; Tables 1-5 | Cancer outcomes, radiation dose estimates, follow-up, and effect measures are extracted. |
| 10b | Data items: variables | Section 2.5; Tables 1-5 | Study characteristics, demographics, CT type/number, dose estimates, follow-up, and risk estimates are reported. |
| 11 | Study risk of bias assessment | Section 2.6; Supplementary Table S1 | Newcastle-Ottawa Scale assessment is reported for each included cohort study. |
| 12 | Effect measures | Section 2.5; Table 3; Results | ERR, RR, HR, IRR, and SIR are identified and interpreted as non-interchangeable measures. |
| 13a | Synthesis methods: eligibility for synthesis | Section 2.7 | Qualitative synthesis is described; formal meta-analysis was not performed because of heterogeneity. |
| 13b | Synthesis methods: preparation | Section 2.7; Tables 1-5 | Studies are grouped by characteristics, exposure patterns, outcomes, dose estimates, and risk measures. |
| 13c | Synthesis methods: tabulation | Tables 1-5 | Study characteristics, CT distribution, risk estimates, cancer cases, and dose estimation methods are tabulated. |
| 13d | Synthesis methods: study results | Results; Discussion | Dose-response, age, cumulative exposure, latency, indication bias, and absolute risk are synthesized narratively. |
| 13e | Synthesis methods: heterogeneity | Section 2.7; Discussion; Limitations | Heterogeneity in design, organ dose, outcomes, latency, comparators, and effect measures is discussed. |
| 13f | Sensitivity analyses | Discussion | No new sensitivity analysis was performed; original-study lag periods and sensitivity approaches are discussed narratively. |
| 14 | Reporting bias assessment | Limitations | Formal publication-bias assessment was not possible because of the small number of comparable estimates. |
| 15 | Certainty assessment | Section 2.6; Discussion; Limitations | NOS assessment and methodological limitations are used to inform certainty qualitatively. |
| 16a | Study selection: search results | Figure 1; Section 2.4 | PRISMA flow diagram and text report record identification, full-text assessment, exclusions, and included studies. |
| 16b | Study selection: excluded studies | Section 2.4; Figure 1 | Full-text exclusion reasons are stated in the Study Selection subsection and summarized in Figure 1. |
| 17 | Study characteristics | Section 3.1; Table 1 | Characteristics of the seven included cohort studies are presented. |
| 18 | Risk of bias in studies | Section 2.6; Supplementary Table S1 | NOS domain scores and overall judgments are presented. |
| 19 | Results of individual studies | Tables 1-5; Results | Risk estimates and cancer cases are reported by study where available. |
| 20a | Results of syntheses | Results; Discussion | Narrative synthesis reports dose-response, age effects, cumulative exposure, latency, and indication bias. |
| 20b | Statistical synthesis | Section 2.7 | No pooled effect estimate was calculated because meta-analysis was judged inappropriate. |
| 20c | Heterogeneity results | Section 2.7; Discussion | Heterogeneity is described qualitatively rather than through pooled statistical measures. |
| 20d | Sensitivity results | Discussion | Original lag-period and indication-bias approaches are discussed where reported by included studies. |
| 21 | Reporting biases | Limitations | Potential publication bias and database restriction are acknowledged. |
| 22 | Certainty of evidence | Discussion; Limitations | Certainty is discussed qualitatively considering NOS, heterogeneity, residual confounding, and reverse causation. |
| 23a | Discussion: interpretation | Discussion | Findings are interpreted in relation to pediatric CT risk, absolute risk, LNT, and clinical benefit. |
| 23b | Discussion: limitations of evidence | Discussion; Limitations | Residual confounding, reverse causation, dose-estimation uncertainty, and follow-up variability are discussed. |
| 23c | Discussion: limitations of review process | Limitations | Database restriction, non-registration, lack of grey literature, and inability to assess publication bias are acknowledged. |
| 23d | Discussion: implications | Discussion; Conclusion | Implications for justification, dose optimization, and non-ionizing alternatives are stated. |
| 24a | Registration and protocol | Section 2.1; Limitations | Protocol was not prospectively registered in PROSPERO; this is stated as a limitation. |
| 24b | Protocol access | Section 2.1; Limitations | No registered protocol is available. |
| 24c | Protocol amendments | Section 2.1 | Not applicable because no registered protocol was available. |
| 25 | Support | Funding statement | Funding statement reports no external funding. |
| 26 | Competing interests | Conflicts of Interest | Authors declare no conflicts of interest. |
| 27 | Availability of data, code, and materials | Data Availability Statement | Review data are derived from publicly available published studies. |
